# An algorithm for the reconstruction of high-energy neutrino-induced particle showers and its application to the ANTARES neutrino telescope

**DOI:** 10.1140/epjc/s10052-017-4979-2

**Published:** 2017-06-21

**Authors:** A. Albert, M. André, M. Anghinolfi, G. Anton, M. Ardid, J.-J. Aubert, T. Avgitas, B. Baret, J. Barrios-Martí, S. Basa, V. Bertin, S. Biagi, R. Bormuth, S. Bourret, M. C. Bouwhuis, R. Bruijn, J. Brunner, J. Busto, A. Capone, L. Caramete, J. Carr, S. Celli, T. Chiarusi, M. Circella, J. A. B. Coelho, A. Coleiro, R. Coniglione, H. Costantini, P. Coyle, A. Creusot, A. Deschamps, G. De Bonis, C. Distefano, I. Di Palma, A. Domi, C. Donzaud, D. Dornic, D. Drouhin, T. Eberl, I. El Bojaddaini, D. Elsässer, A. Enzenhöfer, I. Felis, F. Folger, L. A. Fusco, S. Galatà, P. Gay, V. Giordano, H. Glotin, T. Grégoire, R. Gracia Ruiz, K. Graf, S. Hallmann, H. van Haren, A. J. Heijboer, Y. Hello, J. J. Hernández-Rey, J. Hößl, J. Hofestädt, C. Hugon, G. Illuminati, C. W. James, M. de Jong, M. Jongen, M. Kadler, O. Kalekin, U. Katz, D. Kießling, A. Kouchner, M. Kreter, I. Kreykenbohm, V. Kulikovskiy, C. Lachaud, R. Lahmann, D. Lefèvre, E. Leonora, M. Lotze, S. Loucatos, M. Marcelin, A. Margiotta, A. Marinelli, J. A. Martínez-Mora, R. Mele, K. Melis, T. Michael, P. Migliozzi, A. Moussa, E. Nezri, M. Organokov, G. E. Păvălaş, C. Pellegrino, C. Perrina, P. Piattelli, V. Popa, T. Pradier, L. Quinn, C. Racca, G. Riccobene, A. Sánchez-Losa, M. Saldaña, I. Salvadori, D. F. E. Samtleben, M. Sanguineti, P. Sapienza, F. Schüssler, C. Sieger, M. Spurio, Th. Stolarczyk, M. Taiuti, Y. Tayalati, A. Trovato, D. Turpin, C. Tönnis, B. Vallage, V. Van Elewyck, F. Versari, D. Vivolo, A. Vizzoca, J. Wilms, J. D. Zornoza, J. Zúñiga

**Affiliations:** 1 GRPHE, Université de Haute Alsace, Institut universitaire de technologie de Colmar, 34 rue du Grillenbreit, BP 50568, 68008 Colmar, France; 2 Laboratory of Applied Bioacoustics, Rambla Exposició, Technical University of Catalonia, 08800 Vilanova i la Geltrú, Barcelona, Spain; 3 INFN-Sezione di Genova, Via Dodecaneso 33, 16146 Genoa, Italy; 4 Erlangen Centre for Astroparticle Physics, Friedrich-Alexander-Universität Erlangen-Nürnberg, Erwin-Rommel-Str. 1, 91058 Erlangen, Germany; 5 Institut d’Investigació per a la Gestió Integrada de les Zones Costaneres (IGIC), Universitat Politècnica de València, C/Paranimf 1, 46730 Gandia, Spain; 6 Aix Marseille Univ, CNRS/IN2P3, CPPM, Marseille, France; 7 APC, Univ Paris Diderot, CNRS/IN2P3, CEA/Irfu, Obs de Paris, Sorbonne Paris Cité, Paris, France; 8 IFIC, Instituto de Física Corpuscular (CSIC-Universitat de València) c/Catedrático José Beltrán, 2, 46980, Paterna Valencia, Spain; 9 LAM, Laboratoire d’Astrophysique de Marseille, Pôle de l’Étoile Site de Château-Gombert, rue Frédéric Joliot-Curie 38, 13388 Marseille Cedex 13, France; 10 INFN, Laboratori Nazionali del Sud (LNS), Via S. Sofia 62, 95123 Catania, Italy; 11 Nikhef, Science Park, Amsterdam, The Netherlands; 12 Huygens-Kamerlingh Onnes Laboratorium, Universiteit Leiden, Leiden, The Netherlands; 13 Universiteit van Amsterdam, Instituut voor Hoge-Energie Fysica, Science Park 105, 1098 XG Amsterdam, The Netherlands; 14 INFN, Sezione di Roma, P.le Aldo Moro 2, 00185 Rome, Italy; 15 Dipartimento di Fisica dell’Università La Sapienza, P.le Aldo Moro 2, 00185 Rome, Italy; 16 Institute for Space Science, 077125, Bucharest, Măgurele, Romania; 17 Gran Sasso Science Institute, Viale Francesco Crispi 7, 00167 L’Aquila, Italy; 18 INFN, Sezione di Bologna, Viale Berti-Pichat 6/2, 40127 Bologna, Italy; 19 INFN, Sezione di Bari, Via E. Orabona 4, 70126 Bari, Italy; 20 Géoazur, UCA, CNRS, IRD, Observatoire de la Côte d’Azur, Sophia Antipolis, France; 21 Dipartimento di Fisica dell’Università, Via Dodecaneso 33, 16146 Genoa, Italy; 22 Université Paris-Sud, 91405 Orsay Cedex, France; 23 Laboratory of Physics of Matter and Radiations, University Mohammed I, B.P.717, 6000 Oujda, Morocco; 24 Institut für Theoretische Physik und Astrophysik, Universität Würzburg, Emil-Fischer Str. 31, 97074 Würzburg, Germany; 25 Dipartimento di Fisica e Astronomia dell’Università, Viale Berti Pichat 6/2, 40127 Bologna, Italy; 26 Laboratoire de Physique Corpusculaire, Clermont Université, Université Blaise Pascal, CNRS/IN2P3, BP 10448, 63000 Clermont-Ferrand, France; 27 INFN, Sezione di Catania, Viale Andrea Doria 6, 95125 Catania, Italy; 28 LSIS, Aix Marseille Université CNRS ENSAM LSIS UMR 7296, 13397 Marseille, France; 29 Université de Toulon CNRS LSIS UMR 7296, 83957 La Garde, France; 30 Institut Universitaire de France, 75005 Paris, France; 31 Royal Netherlands Institute for Sea Research (NIOZ), Landsdiep 4, 1797 SZ ’t Horntje (Texel), The Netherlands; 32 Dr. Remeis-Sternwarte and ECAP, Universität Erlangen-Nürnberg, Sternwartstr. 7, 96049 Bamberg, Germany; 33 Moscow State University, Skobeltsyn Institute of Nuclear Physics, Leninskie gory, 119991 Moscow, Russia; 34 Mediterranean Institute of Oceanography (MIO), Aix-Marseille University, 13288 Marseille Cedex 9, France; 35 Université du Sud Toulon-Var, CNRS-INSU/IRD UM 110, 83957 La Garde Cedex, France; 36 Dipartimento di Fisica ed Astronomia dell’Università, Viale Andrea Doria 6, 95125 Catania, Italy; 37 Direction des Sciences de la Matière, Institut de recherche sur les lois fondamentales de l’Univers, Service de Physique des Particules, CEA Saclay, 91191 Gif-sur-Yvette Cedex, France; 38 INFN, Sezione di Pisa, Largo B. Pontecorvo 3, 56127 Pisa, Italy; 39 Dipartimento di Fisica dell’Università, Largo B. Pontecorvo 3, 56127 Pisa, Italy; 40 INFN, Sezione di Napoli, Via Cintia, 80126 Naples, Italy; 41 Dipartimento di Fisica dell’Università Federico II di Napoli, Via Cintia, 80126 Naples, Italy; 42 Université de Strasbourg, CNRS, IPHC UMR 7178, 67000 Strasbourg, France; 43 University Mohammed V in Rabat, Faculty of Sciences, 4 av. Ibn Battouta, B.P. 1014, R.P. 10000 Rabat, Morocco

## Abstract

A novel algorithm to reconstruct neutrino-induced particle showers within the ANTARES neutrino telescope is presented. The method achieves a median angular resolution of $6^\circ$ for shower energies below 100 TeV. Applying this algorithm to 6 years of data taken with the ANTARES detector, 8 events with reconstructed shower energies above 10 TeV are observed. This is consistent with the expectation of about 5 events from atmospheric backgrounds, but also compatible with diffuse astrophysical flux measurements by the IceCube collaboration, from which 2–4 additional events are expected. A $90\%$ C.L. upper limit on the diffuse astrophysical neutrino flux with a value per neutrino flavour of $\text {E}^2\cdot \Phi ^{90\%} = 4.9 \cdot 10^{-8}\,\mathrm {GeV} \cdot \mathrm {cm^{-2} \cdot s^{-1}\cdot sr^{-1}}$ is set, applicable to the energy range from 23 TeV to 7.8 PeV, assuming an unbroken $\text {E}^{-2}$ spectrum and neutrino flavour equipartition at Earth.

## Introduction

With the discovery of a diffuse astrophysical neutrino flux by the IceCube observatory located in the deep Antarctic ice, high-energy neutrino astronomy has reported its first observation [1–3]. The extraterrestrial origin of the flux has been established with high significance [4–6]. Although the sources of these high-energy neutrinos have not yet been pinned down, it is expected that their identification will help to elucidate the sites and mechanisms of baryonic acceleration, and will play a key role in the discovery of the sources of Galactic and extragalactic cosmic rays.

In neutrino telescopes in ice or water, a charged-current (CC) interaction of a $\upnu _{\upmu }$ or $\overline{\upnu }_{\upmu }$ (in the following abbreviated to ) inside or around the instrumented volume creates a relativistic muon whose long trajectory can, depending on its energy, cross the entire detector and be detected by photomultipliers (PMTs) through the induced Cherenkov light emission. The event signature due to neutral-current (NC), and  and  CC interactions inside or close to the instrumented volume is however a particle shower^1^ (also often referred to as a shower-like or cascade event) with a characteristic longitudinal extension of a few meters that increases logarithmically with energy. The particle shower constitutes a Cherenkov light source which appears localised compared to the typical distances between photosensors in neutrino telescopes. This light emission characteristic offers the opportunity to estimate the energy released in a neutrino-induced shower more reliably than that of muons, while the direction determination is more difficult and generally results in a worse angular resolution.

A high-energy astrophysical neutrino flux has been observed and characterised in several different analyses by IceCube. The high-energy starting event analysis identifies neutrino-interaction vertices of all flavours contained in the detector volume. In 4 years of data taking, it has observed 54 events from the entire sky, of which 39 have been identified as shower-like with a typical directional resolution of about $15^{\circ }$ [5]. A best-fit spectral index of $\Gamma =2.58\pm 0.25$ is obtained, assuming a power-law flux model $\text {dN}_{\upnu }/\text {dE}_{\upnu } = \Phi _0\text {E}^{-\Gamma }$. The flux normalisation at 100 TeV of $\Phi _0 = 2.2\times 10^{-8}\,\mathrm {GeV} \cdot \mathrm {cm^{-2} \cdot s^{-1}\cdot sr^{-1}}$ is valid per neutrino flavour, and for neutrinos yielding a deposited energy between $60\,\text {TeV}$ and $3\,\text {PeV}$. Recently, a complementary measurement of an astrophysical neutrino flux has been achieved using only CC muon neutrino events from the Northern sky. Using 6 years of data, an astrophysical flux with a hard spectral index of $\Gamma =2.13\pm 0.13$ and a normalisation at 100 TeV of $\Phi _0 = 0.9\times 10^{-8}\,\mathrm {GeV} \cdot \mathrm {cm^{-2} \cdot s^{-1}\cdot sr^{-1}}$ has been found for neutrino energies above roughly 200 TeV [6]. This result shows a $3.3\,\upsigma$ tension with the normalisation value and soft spectral index obtained in a fit combining different previous IceCube analyses with mainly lower energy thresholds [8], which could be indicative of a spectral break [6]. The measurements indicate that a substantial fraction of the flux must be of extragalactic origin, while a Galactic contribution could be the reason for the observed tension. Exploiting the limited statistics of the available astrophysical neutrino sample, first indications have been put forward that the observed flux is anisotropic, being slightly stronger and exhibiting a softer spectrum in the region of the Galaxy in the Southern sky [9, 10]. The $\upnu _{\text {e}}{:} \upnu _{\upmu }{:} \upnu _{\uptau }$ ratio is compatible with 1:1:1 [8], consistent with expectations from charged meson decays in cosmic-ray accelerators and 3-flavour neutrino mixing. Dedicated searches for small-scale anisotropies in neutrino arrival directions and for spatial correlations with known astrophysical sources have not revealed statistically significant deviations from the isotropy hypothesis [11–14].

Given the tensions and uncertainties in the observations by IceCube, it is important to provide additional measurements and complementary sky coverage in the track-like muon neutrino and in the shower-like all-flavour event channels. ANTARES is a neutrino telescope located in the Northern Hemisphere which, despite having a significantly smaller volume than IceCube, has a comparable muon neutrino effective area at TeV energies for observations of the Southern sky [12]. ANTARES data have been used to set constraints on, e.g., the all-sky diffuse muon neutrino flux [15, 16], the strength of a possible Galactic component of the flux discovered by IceCube [17], and the possible neutrino flux from the region of the Galactic Ridge [18]. Furthermore, several searches for clustering and large-scale anisotropies in the neutrino arrival directions, as well as for temporal and/or spatial correlations with known astrophysical sources have been carried out [19–22].

This paper presents a reconstruction algorithm for neutrino-induced particle shower events and reports on the first application of such an algorithm to ANTARES data. The reconstruction method has been employed to search for a diffuse astrophysical neutrino flux using 6 years of data collected from 2007 to 2012. The ANTARES detector is described in Sect. 2. The detector simulation and the developed algorithm are presented in Sects. 3 and 4, respectively. The data selection is discussed in Sect. 5, while the analysis method and the discussion of systematic uncertainties can be found in Sect. 6.

The results of the search are reported in Sect. 7, while Sect. 8 summarizes and concludes the paper. The presented work is used as input to more advanced reconstruction algorithms based on updated simulations which are in development [23].

## The ANTARES neutrino telescope

The ANTARES neutrino telescope [24] is located in the Mediterranean Sea about 40 km offshore from Toulon in a depth of about 2500 m, and comprises a three-dimensional array of 885 PMTs housed inside glass spheres, denoted as optical modules (OMs) [25]. The OMs are attached to 12 readout cables (lines), each holding 75 of these arranged in groups of three on 25 storeys.^2^ The vertical spacing between storeys is 14.5 m, while the horizontal spacing between lines deployed in an approximately octagonal configuration is about 60 m on average. The detector instruments a water mass of roughly $20\,\text {Mt}$, but can be sensitive to neutrino interaction events outside of this volume, depending on the distance of the neutrino interaction point (vertex) to this volume, the neutrino direction and the event light yield. ANTARES is mainly sensitive to neutrinos of TeV to PeV energies, with a threshold for astrophysical studies of roughly $100\,\mathrm {GeV}$.

If the analogue output signal of a PMT reaches an amplitude corresponding to a charge above a tunable threshold of typically 0.3 photoelectrons (pe), the signal time and charge are digitised, and this pair of values is denoted as a “hit” [27]. Events are selected by different triggering algorithms [28] that causally connect hits in time and space. The achieved resolutions on the arrival time of photons at the PMTs, measured with nanosecond precision [29], and on the position and orientation of the OMs [30], as well as the low photon scattering probability in seawater [31], allow for the reconstruction of the triggered events with excellent angular resolution for muon neutrino CC events [32].

Two different types of backgrounds have to be taken into account in the event reconstruction algorithms and in the search for high-energy astrophysical neutrinos. The time variable photon emission by deep-sea bioluminescent organisms and Cherenkov photons induced by electrons from beta decays of radioactive potassium (${}^{40}$K) add PMT hits unrelated to those caused by the detection of Cherenkov photons from the passage of relativistic particles. The second type of background consists of events that are induced by atmospheric neutrinos and muons produced in interactions of cosmic rays with the Earth’s atmosphere. Using the Earth as a shield against the atmospheric muon background, upward-going neutrinos are observed that predominantly originate from the Southern sky due to the geographical location of the telescope.

Individual upward-going atmospheric neutrinos are indistinguishable from neutrinos of astrophysical origin, unless observed in temporal and/or spatial coincidence with other cosmic messengers [33, 34].

## Simulation of signal and background

For the development of the shower reconstruction algorithm and for the optimisation of the diffuse neutrino flux search, detailed Monte-Carlo (MC) simulations of the detector response to both signal and background events are used [35, 36].

Some of the deep-sea environmental conditions typically change on a timescale of a few hours. In particular, the optical background rates, which are measured for each OM individually, can show significant variations with time, and are of relevance for the data acquisition and the detector efficiency. In order to take these variations into account, each data-taking period of a few hours (denoted as a *run*) is simulated individually [37]. The background is generated according to the measured rates on each active OM, which are determined with a sampling frequency of roughly 10 Hz. Additionally, PMT individual charge calibrations and effective thresholds are used, and the simulated hit time and charge is smeared. Finally, the simulated events are processed with the same trigger algorithms active during data acquisition.

The generation of  and  neutrino interactions is performed using the LEPTO [38] package for deep inelastic scattering processes and RSQ [39] for resonant and quasi-elastic processes using the CTEQ6-DIS [40] parton distribution functions. The hadronisation is performed using PYTHIA/JETSET [41]. Interactions of  are not simulated and their contribution is estimated differently, as discussed in Sect. 6. In order to obtain sufficient statistics at high energies, the  and  events are generated with a hard $\mathrm {E}^{-1.4}$ spectrum. A reweighting procedure is employed to simulate different astrophysical and atmospheric neutrino flux models from the generated events.

The generation of atmospheric muon events uses the MUPAGE [42, 43] package. The propagation of muons in water is achieved with MUSIC [44]. For muon events, no reweighting procedure is used, but an integrated flux corresponding to one third of the data-taking livetime is generated.

For hadronic showers induced by neutrinos with an energy below $100\,\mathrm {TeV}$, each particle generated in the interaction and its corresponding light emission is simulated with GEANT 3.21 [45]. Electromagnetic showers and their photon emission are generated using parametrisations and precomputed probability tables. For neutrino events with energies above $100\,\mathrm {TeV}$, hadronic showers are simulated using a one-particle approach, i.e. all hadrons are replaced with an equivalent electron whose energy is determined from that of the hadrons by an appropriate weighting scheme.

In order to keep the computational cost of the simulation manageable, two additional simplifications are introduced. For photons generated in particle showers, scattering processes are not taken into account, and for  CC events with $\mathrm {E}_{\upnu } > 100\,\text {TeV}$, Cherenkov photon emission from the hadronic vertex shower is not simulated. Both simplifications are taken into account in the analysis by corrections and corresponding systematic uncertainties, which are derived from dedicated simulations and discussed in Sect. 6.

## Shower event reconstruction

For the selection and reconstruction of triggered events that contain a shower, a dedicated maximum-likelihood-based reconstruction algorithm has been developed. It allows for the estimation of the shower energy, of the interaction point and time, and of the direction of the incoming neutrino.

In a pre-fit step, the shower position and time are roughly estimated. To this end, hits caused mainly by unscattered light are selected by considering only the earliest hit on each OM. A $\chi ^2$-fit scanning for the time and position of the shower is done assuming a spherical light source, and using only OMs on storeys with at least two hits within $20\,\mathrm {ns}$. As optical background processes, such as ${}^{40}$K decays or bioluminescence, induce mainly single photoelectron hits, restricting the hit selection to coincidences with a charge exceeding $1.2\,\text {pe}$ per hit ensures that this pre-fit is performed on a sample dominated by signal hits. This signal hit selection has been developed with dedicated simulations including scattering for photons induced by shower particles, and has been verified by comparing measured and simulated hit time distributions.

**Fig. 1:**
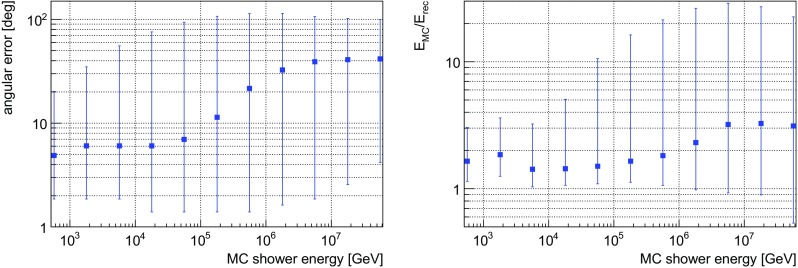
*Left* angular error of the direction reconstruction for shower-like neutrino events as a function of the MC shower energy. *Right* the ratio of the MC and the reconstructed shower energy, as a function of the MC shower energy. *Blue squares* denote the median of the distributions. The *lower* and *upper end* of the *vertical bars* in *both figures* show the 10 and 90% quantiles of the distributions, respectively

In the next step, a new hit selection takes into account all hits in the event again. Hits are selected if their distance to at least one storey with coincident hits or to the shower position estimated in the previous step is lower than $50\,\mathrm {m}$. Additionally, the hit time must be in a range of $\pm 80\,\mathrm {ns}$ with respect to the arrival time expectation assuming isotropic photon emission at the estimated shower position. The chosen value of the distance criterion corresponds roughly to the seawater absorption length [46] and prevents far-away background hits that coincidentally fit to the isotropic light emission hypothesis from being falsely selected. If this procedure finds fewer than 5 hits in total or hits on less than 3 lines, the event is discarded. The remaining contamination from noise-induced hits has been estimated to be about 1%.

Refining the results of the pre-fit and based on this second hit selection, the parameters of the shower are determined with two consecutive maximum-likelihood fits. Both fits make use of precomputed probability tables that have been obtained using the detailed MC simulations described in Sect. 3. The first maximum-likelihood fit determines the position and time of the shower. It varies these shower parameters and evaluates the precomputed probability for each selected hit, given its time and position, to be due to Cherenkov photons emitted at the assumed shower time and position. The second fit determines the direction of the incoming neutrino and the energy of the particle shower resulting from the neutrino interaction, while fixing the start time and position of the shower to the values found by the first fit. This factorisation of the fitting procedure is possible due to the large scattering length of seawater and due to the homogeneity of the medium, which allows for the position reconstruction of the maximum shower light yield independent of the shower direction.^3^ This second fit is based on precomputed and tabulated probabilities for hits to be due to Cherenkov photons emitted in a particle shower with given energy, time and position, and induced by a neutrino with given direction. The three-dimensional probability table depends on the photon emission angle, the total photon yield emitted by the shower, and the energy of the shower. The photon emission angle is defined as the angle between the direction of the incoming neutrino and a straight line from the shower position to the hit OM. The shower charge $\text {c}_{\text {shower}}$, in units of photoelectrons and with typical values of about $10^8$ pe for 10 TeV shower energy, is used as a proxy for the total light yield from the shower and is defined as $\text {c}_{\text {shower}} = \text {c}_{\text {hit}} \cdot \text {e}^{\frac{\text {d}}{\uplambda _{\text {w}}}} \cdot \frac{1}{\upalpha } \cdot \frac{4\uppi \text {d}^2}{\text {A}_{\text {OM}}}$, where $\text {c}_{\text {hit}}$ is the measured charge of the hit with a maximum value of about 25 pe, $\uplambda _{\text {w}}$ is the attenuation length of seawater [31] and $\upalpha$ is the incidence-angle-dependent photon-detection probability of an OM. The last factor relates the OM cross-section $\text {A}_{\text {OM}}$ [25] to the total surface of a sphere defined by the radial distance d of the shower position to the OM. The definition of the parameter $\text {c}_{\text {shower}}$ was chosen to make the shower energy estimate approximately independent of the detected light yield, allowing for the reconstruction of events in which the emitted light partly escapes the sensitive volume of the detector.

In the search for astrophysical neutrinos described later, a quality cut on the likelihood of the vertex fit (*vertex-quality cut*) is applied. It aims at optimising the signal to background ratio by efficiently selecting neutrino-induced shower events while vetoing atmospheric muons. Applying this cut yields a 3 (6) $\mathrm {m}$ median position resolution for the neutrino interaction vertex for events with a MC shower energy of $100\,\mathrm {GeV}$ $(1\,\mathrm {PeV})$. In particular, for high shower energies, this resolution is dominated by the distance between the interaction vertex and the position of the shower light yield maximum. The MC shower energy is defined by the fraction of the neutrino energy deposited at the vertex, thus contributing to the shower light yield. For  CC events, it is equivalent to the neutrino energy, while it is lower by the energy of the escaping neutrino for NC events.

The distribution of the angular error on the neutrino direction in Fig. 1 (left) shows a median value of about $6^\circ$ for shower energies up to $100\,\mathrm {TeV}$, and worsens to about $25^\circ$ ($40^\circ$) at $1\,\mathrm {PeV}$ ($10\,\mathrm {PeV}$). This is a consequence of the stronger light yield at higher energies that saturates the detector and increasingly impedes the efficient recognition of the emission direction of Cherenkov light from the shower particles.

The ratio between the MC and the reconstructed energy $\mathrm {E_{MC}}/\mathrm {E_{rec}}$, characterised by its median value as well as the 10 and 90% quantiles, is depicted as a function of shower energy in Fig. 1 (right). The median value stays below 2 for shower energies up to $1\,\mathrm {PeV}$, and increases to about 3 at $10\,\mathrm {PeV}$. While 90% of the events are reconstructed with a ratio $\mathrm {E_{MC}}/\mathrm {E_{rec}}$ up to 4 for energies below 10 TeV, the distribution widens significantly up to PeV energies, again as a consequence of the light yield saturating the detector.

**Fig. 2:**
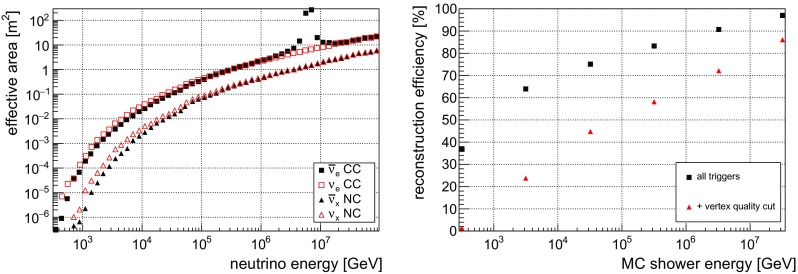
*Left* the neutrino effective area after applying the vertex-quality cut to triggered events, and integrated over all directions, as a function of simulated neutrino energy for $\overline{\upnu }_{\text {e}}$ (*black full squares*) and $\upnu _{\text {e}}$ (*red open squares*) CC events, and $\overline{\upnu }$ (*black triangles*) and for $\upnu$ (*red open triangles*) NC events. *Right* reconstruction efficiency for all triggered shower-like events (*black squares*) and including the vertex-quality cut (*red triangles*) as a function of MC shower energy

The effective area for the detection of  CC and all-flavour NC events after applying the vertex-quality cut is depicted as a function of the simulated neutrino energy in Fig. 2 (left). The peak in the effective area at roughly 6 PeV for $\overline{\upnu }_{\text {e}}$ corresponds to the Glashow resonance [48]. As shown in Fig. 2 (right), the fraction of successfully reconstructed events among all triggered shower-like events increases from 50 to $90\%$ as a function of shower energy in the range from $1\,\mathrm {TeV}$ to $3\,\mathrm {PeV}$. Applying the vertex-quality cut, roughly 10–70% of all triggered shower-like events remain for the same energy range, while the atmospheric muon background is reduced by 5 orders of magnitude. The remaining atmospheric muons are reconstructed with a mean zenith-angle error of about $7^\circ$. Further details can be found in Ref. [49].

## Data selection

The reconstruction algorithm described in the previous section was applied to data collected from February 2007 to December 2012. This includes the construction and commissioning phase of the detector and therefore several detector configurations, each comprising a different number of active lines included in the data taking. All of these configurations have been reproduced by the detailed run-based simulation procedure described in Sect. 3. The data analysis was designed blindly, i.e. the neutrino selection criteria have been developed using the simulations only. A fraction of 10% of the data runs (test data), sampled from the full data collection time range, was compared to simulations to validate the selection criteria. These test data were excluded from the neutrino search described in Sect. 6. Simulation studies, as well as a comparison to the test data, did not reveal any significant influence of the time-variable optical background rates on the performance of the shower reconstruction strategy presented in Sect. 4. This is to be expected, as the typical optical background rates in the ANTARES detector are of the order of 50–80 kHz per PMT, while even for extreme and rare conditions of several hundred kHz, the probability of any given PMT having a background hit in $\pm 80\,\mathrm {ns}$ is of the order of a few percent.

Active PMTs have been observed to occasionally produce a flash of light inside OMs, and photons from this flash are detected by other PMTs in the vicinity. This phenomenon is rare, with only a few occurrences over the whole data-taking period. Runs that have been identified to contain at least one flashing PMT were excluded from the analysis. In order to further suppress this background, events were vetoed if the shower position is reconstructed closer than $15\,\text {m}$ to any of the OMs. This cut (*discharge cut*), which reduces the sensitive volume within the instrumented detector by about 30%, was chosen conservatively after a dedicated analysis of events with flashing PMTs. Note that this cut is not included in the effective area shown in Fig. 2 (left).

Removing the 10% test data, a total effective data-acquisition livetime of 1247 days is included in the analysis.

## Analysis method and systematic uncertainties

The presented analysis used 6 years of ANTARES data to search for an excess over the atmospheric background of upward-going astrophysical neutrinos inducing high-energy showering events.

The method is complementary to the first searches for a diffuse neutrino flux performed with ANTARES [15, 16], which selected only the track-like event signatures of upward-going muons induced by  CC interactions. Even though NC interactions of atmospheric  contribute to the background for the presented search, the small value of the ratio of atmospheric  to  fluxes at TeV energies [54] reduces the overall background compared to the earlier analyses.

We treat the search for astrophysical neutrinos as a simple counting experiment, and derive confidence intervals using the unified approach of Feldman et al. [51]. We optimise the selection criteria for the best upper limit, also known as model rejection factor (MRF) optimisation [50].

Requiring successfully reconstructed shower-like events with hits on at least 3 lines, which survive the vertex-quality (cf. Sect. 4) and the discharge cut (cf. Sect. 5), reduces the atmospheric muon background in the simulated event sample down to about 1000 events, and about 100 (10) atmospheric (cosmic) neutrinos remain in the sample.

Selecting only upward-going shower events by cutting on their reconstructed zenith angle, $\Theta _{\text {rec}}=0^{\circ }$ defines vertically down-going while $\Theta _{\text {rec}}=180^{\circ }$ is straight up-going, reduces this contamination further by a factor of about 50. Cutting on the reconstructed shower energy, $\text {E}_{\text {rec}}$, in principle allows for the discrimination of astrophysical and atmospheric neutrino contributions to the flux, since the energy spectrum of astrophysical neutrinos is expected to be harder than that of atmospheric neutrinos.

The MRF is minimised for a neutrino energy spectrum with spectral index $\Gamma =2.0$ by varying $\text {E}_{\text {rec}}$ and $\Theta _{\text {rec}}$, and the optimum is obtained for $\text {E}_{\text {rec}}\ge 10\,\text {TeV}$ and $\Theta _{\text {rec}} \ge 94^\circ$. It is found that this cut combination vetoes the last simulated atmospheric muon events, and that it is largely independent of the exact spectral shape of the neutrino signal, in particular for softer spectral indices. With these cuts applied, the simulations yield an expectation of 1.3 to 2.9 signal events () from a diffuse astrophysical flux with the spectral index and normalisation as reported by IceCube in Refs. [5, 6], respectively.

In the following, all reported event contributions are given for the cut level after the MRF optimisation. From the simulated atmospheric background, 2.3 events are expected using the Bartol atmospheric neutrino flux model [52] and 0.3 events from the prompt atmospheric neutrino component. The latter assumes a flux corresponding to the upper limit determined in Ref. [6], i.e. 50% of the flux predicted in Ref. [53]. As no simulated atmospheric muon remains, the residual contamination of atmospheric muons reconstructed as upward-going showers is estimated by an extrapolation scheme. The efficiency of the vertex-quality cut applied on the sample of events that survive the energy and zenith-angle cut was evaluated as a function of the vertex-quality cut and was extrapolated to the strict cut value used for the final event selection. The validity of this extrapolation scheme has been confirmed with looser cuts on the zenith angle which allowed to compare with the number of muons remaining in the sample. This yields an estimate on the remaining atmospheric muon contribution of 1.8 events after the final cuts.

The contribution from astrophysical  was estimated assuming flavour equipartition at Earth for the astrophysical neutrino signal. In the NC channel,  interactions are assumed to create showers identical to those of  and  interactions. The contribution of  CC interactions was estimated from the  channel, taking into account that a fraction of $82.6\%$ of all created $\uptau ^{\pm }$ leptons will give rise to particle showers through their decay. This procedure estimates a total astrophysical  contribution of 0.5 to 1.2 events for the fluxes in Refs. [5, 6], with an uncertainty of about 30%, taking into account that the $\uptau ^{\pm }$ track length before decay exceeds the median vertex resolution of the presented reconstruction for $\uptau ^{\pm }$ energies above roughly $100\,\mathrm {TeV}$, and can thus affect the shower fit. The contribution of prompt atmospheric  is negligible [53].

**Table 1:** Event number expectations corresponding to 1247 days of data taking for the diffuse neutrino flux search derived from simulations for signal and background events. The range for the astrophysical event numbers corresponds to the fluxes as reported in Refs. [5, 6], respectively. Event numbers for a given neutrino flavour denote the sum of neutrinos and their respective antineutrinos. Additionally, the assumed systematic uncertainties on the fluxes, and uncertainties on the detection efficiency, as inferred from detector simulations after the vertex-quality cut only (cf. Sect. 4), are shown

Events selected by final cuts		Syst. uncertainties	
Type	Number	Flux	Detection
Conventional atmospheric	2.3	±30%	${}^{+17}_{-23}\%$
+ hadr. vertex corr. for $\text {E}_{\upnu _{\upmu }}>100$ TeV	$\le$0.2		
Prompt atmospheric	0.3	${}^{+25}_{-40}\%$	–
Atmospheric $\upmu$	1.8	±30%	${}^{+21}_{-22}\%$
Astrophysical	1.3–2.9	–	${}^{+14}_{-10}\%$
+ hadr. vertex corr. for $\text {E}_{\upnu _{\upmu }}>100$ TeV	$\le$0.3		
Astrophysical	0.5–1.2	–	±30%

For  CC events with $\mathrm {E}_{\upnu } > 100\,\text {TeV}$, photon emission from the hadronic vertex shower has not been simulated, cf. Sect. 3. A dedicated analysis of the reconstructed energy spectrum of such events for energies above and below $100\,\text {TeV}$ was used to quantify their additional contribution to the sample of reconstructed shower events. This estimate yields a small additional contribution of at most 0.3 (0.2) events from the astrophysical (atmospheric)  flux.

The systematic uncertainty on the normalisation of the conventional atmospheric neutrino flux was assumed to be $\pm 30\%$ [54, 55]. The same was assumed as the relative uncertainty on the number of atmospheric muons. The parametrisation in Ref. [53] was employed for the prompt atmospheric neutrino flux which yields on average an uncertainty of ${}^{+25}_{-40}\%$.

The influence of the uncertainty on the light absorption length and the scattering length of seawater, and on the average PMT efficiency has been determined by varying the nominal parameter values in the detector simulation independently by $\pm 10$% [56]. The resulting individual uncertainties for the event detection efficiencies were added in quadrature. The number of simulated events surviving all cuts relevant for the diffuse neutrino flux search, the assumed uncertainties on the respective fluxes and the detection uncertainties for the different fluxes are summarised in Table 1. Neutrino events generated according to a hard astrophysical spectrum are on average more energetic and hence induce a larger number of signal hits in the detector compared to atmospheric neutrino events, and their respective detection uncertainties are therefore smaller.

**Fig. 3:**
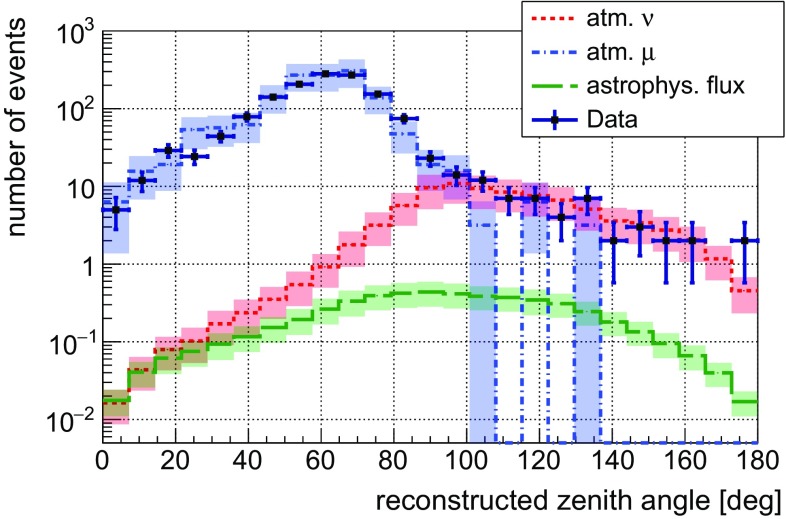
Reconstructed zenith-angle distribution for 1247 days of data taking, with events selected as described in Sects. 4 and 5. Data points and their statistical errors are depicted with *black markers* and compared to simulated distributions of atmospheric muons (*blue*), atmospheric neutrinos (*red*) and the astrophysical flux reported in Ref. [6] (*green*). The *coloured bands* indicate the uncertainties on the simulated and measured flux normalisations

The uncertainty induced by the missing photon scattering in the simulation of shower events has been investigated by a dedicated simulation including photon scattering processes. It was found that on average $30\%$ less shower events with simulated photon scattering survive the vertex-quality cut, which is taken into account as a systematic uncertainty on the number of shower events in the following.

## Results

Summing up the discussed atmospheric background contributions and correction estimates (cf. Table 1), $\text {n}_{\text {b}}=4.6^{+2.8}_{-3.0}$ background events are expected. For the full dataset of 1247 days, this analysis yields a sensitivity to an astrophysical neutrino flux of:

$$\begin{aligned} \text {E}^2 \cdot \bar{\Phi }^{\text {90}\%} = 2.2^{+0.9}_{-0.7} \cdot 10^{-8} \, \mathrm {GeV} \cdot \mathrm {cm^{-2} \cdot s^{-1}\cdot sr^{-1}}\end{aligned}$$

per flavour, assuming an unbroken $\text {E}^{-2}$ power law spectrum and flavour equipartition at Earth.

Figure 3 shows the reconstructed zenith-angle distribution. The cuts discussed in Sects. 4 and 5 were applied. The measured distribution compares well to the MC expectations from the atmospheric muon and neutrino backgrounds. The zenith-angle distribution of the atmospheric neutrino background is asymmetric with respect to the horizon, which results from the convolution of the assumed atmospheric neutrino flux model [52] and the detector acceptance.

Applying a cut on the reconstructed zenith angle $\Theta _{\text {rec}} \ge 94^\circ$, as derived in the MRF optimization procedure, 60 upward-going events remain, while 35 have a reconstructed shower position inside the instrumented volume. As expected from simulations, the remaining are reconstructed at a maximum distance of $84\,\mathrm {m}$ to the surface of the volume enclosed by the detector lines.

**Fig. 4:**
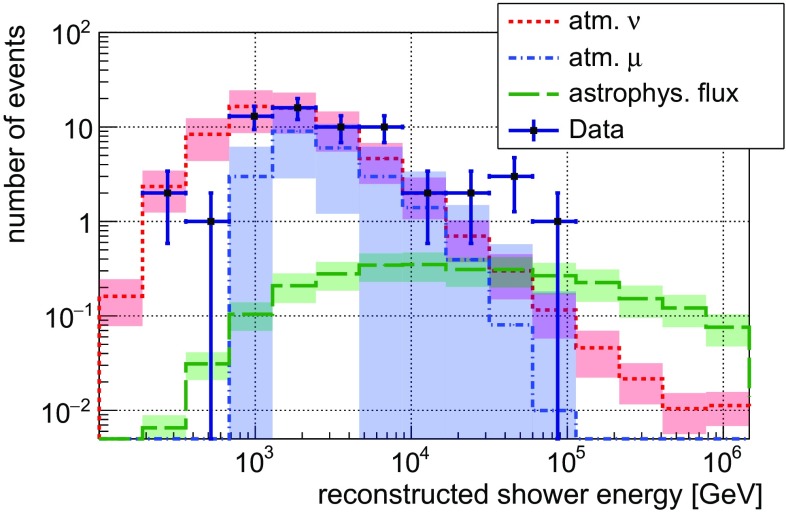
Distribution of the reconstructed shower energy for 1247 days of data taking, selected as described in Sect. 4 and with a cut on the reconstructed zenith angle applied at $\Theta _{\text {rec}} \ge 94^\circ$ (*black markers*, statistical errors only). Simulated contributions from atmospheric muons (*blue*), atmospheric neutrinos (*red*) and an astrophysical flux  [6] (*green*) have been overlaid for comparison. *Coloured bands* indicate the uncertainties on the simulated and measured flux normalisations. The atmospheric muon contribution beyond 10 TeV has been extrapolated as described in Sect. 6

Figure 4 depicts the reconstructed energy spectrum of these 60 events, again compared to expectations derived from simulations. Applying the additional and final cut on the reconstructed shower energy $\text {E}_{\text {rec}}\ge 10\,\mathrm {TeV}$ results in 8 remaining events. All of these events have their shower vertex position reconstructed outside of the instrumented volume. Each of these 8 events has been investigated individually by a dedicated event-based MC simulation. One event was identified to have surpassed the $\ge$3 line veto criterion (cf. Sect. 4) due to 2 additional, isolated random hits on 2 different lines which coincidentally matched to the shower hypothesis. This is a scenario which is in principle covered by the run-based simulation concept that accounts for the OM-individual background rates at the time of the data taking. The remaining 7 events could be verified to have a reconstruction error comparing well to the resolutions discussed in Sect. 4.

Using Poisson statistics, the observation of 8 events with an expectation of 4.6 corresponds to an excess with a significance of $1.6\,\upsigma$. This result agrees with the assumption of a purely atmospheric origin of the observed events, but it is also compatible with the expectations from the diffuse astrophysical neutrino fluxes as reported by the IceCube collaboration.

Following the Feldman-Cousins approach [51] a $90\%$ C.L. upper limit on the number of signal events of $\upmu _{\text {90}\%} = 9.1$ is evaluated from the 8 measured and $\text {n}_{\text {b}}=4.6^{+2.8}_{-3.0}$ expected background events. Systematic uncertainties (including that arising from the missing photon scattering in our simulation, cf. Sect. 6) have been taken into account following the method detailed in Refs. [57, 58].

The relative uncertainties on the signal and background efficiencies, calculated as the average of their systematic error intervals, are evaluated to $29\%$ for the astrophysical signal and $42\%$ for the atmospheric background. This increases the 90% C.L. upper limit of the confidence interval to 11.4 events. For the unblinded data set of 1247 days, the upper limit on the diffuse astrophysical neutrino flux per neutrino flavour is then evaluated to:

$$\begin{aligned} \text {E}^2 \cdot \Phi ^{90\%} = 4.9 \cdot 10^{-8} \, \mathrm {GeV} \cdot \mathrm {cm^{-2} \cdot s^{-1}\cdot sr^{-1}}. \end{aligned}$$

The limit is valid under the assumption of flavour equipartition at Earth and for an unbroken $\text {E}^{-2}$ spectrum in the energy range from $23\,\mathrm {TeV}$ to $7.8\,\mathrm {PeV}$. This range was obtained from the simulated neutrino energy spectrum of all astrophysical shower-like events by determining its central 90% interval.

## Summary and conclusion

A novel event reconstruction algorithm has been presented, which allowed for the first time to select and reconstruct particle-induced shower events in data taken with the ANTARES neutrino telescope. The algorithm achieves a median angular resolution of $6^\circ$ for shower energies below 100 TeV. The median value of the true over reconstructed shower energy ratio, $\mathrm {E_{MC}}/\mathrm {E_{rec}}$, is 1.5–2 for shower energies up to 1 PeV, while the 90% quantile increases from 3 to 20 for energies between 500 GeV and 1 PeV. The fraction of successfully reconstructed events among all triggered shower-like events increases from 50 to $90\%$ as a function of the shower energy in the range from 1 TeV to 3 PeV.

Using 1247 days of ANTARES data, a $90\%$ C.L. upper limit on a diffuse astrophysical neutrino flux per flavour was evaluated to:

$$\begin{aligned} \text {E}^2 \cdot \Phi ^{90\%} = 4.9 \cdot 10^{-8} \, \mathrm {GeV} \cdot \mathrm {cm^{-2} \cdot s^{-1}\cdot sr^{-1}}. \end{aligned}$$

The limit is valid in the energy range from $23\,\mathrm {TeV}$ to $7.8\,\mathrm {PeV}$, assuming an unbroken $\text {E}^{-2}$ neutrino spectrum and flavour equipartition at Earth. It has been calculated using the Feldman-Cousins approach [51]. Systematic errors have been taken into account following Refs. [57, 58].

**Fig. 5:**
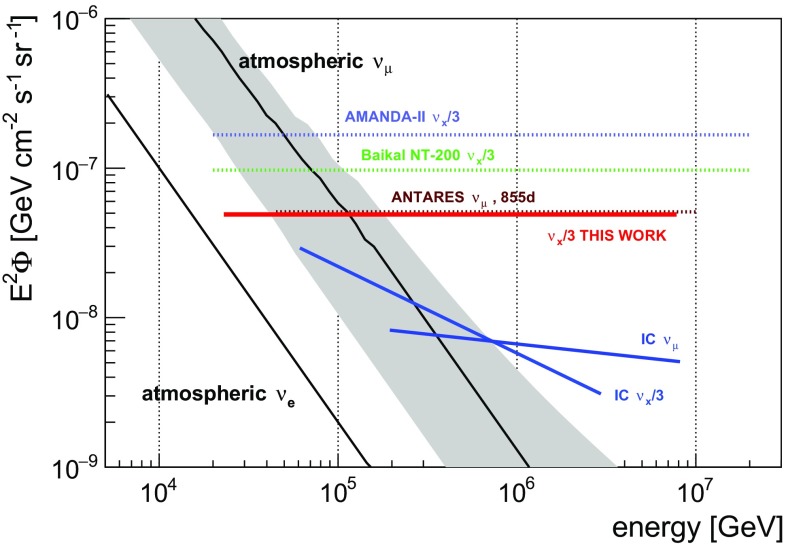
The 90% C.L. upper limit on the diffuse all-flavour astrophysical neutrino flux obtained in this work (*solid red line*) in comparison to previously set upper limits (*dotted lines*, AMANDA-II  [59], Baikal NT-200 [60], and ANTARES $\upnu _{\upmu }$ [16]) and 2 different measurements of a diffuse astrophysical neutrino flux reported by IceCube (*solid blue lines*, IC $\upnu _{\text {x}}/3$  [5], and IC $\upnu _{\upmu }$ [6])

Figure 5 illustrates the obtained upper limit in comparison with previously set limits by the AMANDA [59] and Baikal [60] experiments. The upper limit obtained in this work almost coincides with those obtained previously with ANTARES, using only upward-going muons recorded in 855 [16] and 334 [15] days, although the sensitivity of the present dataset is about a factor of two and three better, respectively. Also shown are the two most recent IceCube measurements of an astrophysical flux that have been obtained either with analyses selecting contained events [5] or using through-going muon tracks originating from the Northern sky [6]. All flux limits and measurements are given per flavour and represent the sum of neutrino and antineutrino fluxes. For comparison, the conventional atmospheric $\upnu _{\upmu }$ flux (black solid line with the gray shaded area showing systematic uncertainty) according to the Bartol neutrino flux model [52] and the measured atmospheric $\upnu _{\text {e}}$ flux [61] is indicated.

The reported measurement of 8 events is statistically in agreement with the expected background of $4.6^{+2.8}_{-3.0}$ events from atmospheric muons and neutrinos. Assuming an astrophysical flux as reported in Ref. [6] ([5]), additional 2.1 (4.4) signal events are expected, which reduces to 1.7 (4.2) events assuming a cut-off at 3 PeV. In all cases, the addition of an astrophysical neutrino signal is compatible with our measurement.

Though not yet sufficiently sensitive, the presented first shower analysis using the initial 6 years of data taken with the ANTARES neutrino telescope demonstrates the potential of ANTARES to independently confirm and complement the measurement of a high-energy astrophysical neutrino flux, as performed by IceCube. In order to meet this important goal, several improvements of the analysis have been identified and are under way. Building on the gained experience, a second shower reconstruction strategy is developed. It improves on the angular resolution and increases the shower event selection efficiency, while continuing to provide the necessary strong suppression of the atmospheric muon background. Using the track reconstruction already employed in our previous searches for a diffuse flux with muon neutrinos [15, 16], an analysis combining both track-like and shower-like events is in progress. With the addition of the remaining ANTARES data until the scheduled end of its operation time in 2017, this combined search is expected to reach a sensitivity at the level of the flux discovered by IceCube [4].

## Footnotes

1 With the exception of about 17% of tau leptons decaying to muons that appear as track-like events [7].

2 The 12th line holds only 20 storeys with OMs. The remaining storeys house a test system for acoustic neutrino detection [26].

3 cf. Ref. [47], in particular Sec. 4.4.2.
